# Ergonomic posture breaks enhance solute transport in cartilage: a musculoskeletal–poroelastic modelling study

**DOI:** 10.1007/s00421-026-06173-1

**Published:** 2026-03-07

**Authors:** Hanneng Guo, Qianjun Ding, Saeed Miramini, Lihai Zhang

**Affiliations:** Department of Infrastructure Engineering, The University of Melbourne, Melbourne, VIC 3010 Australia

**Keywords:** Insulin-like growth factor 1 (IGF-1), Cartilage, Solute transport, Workplace ergonomics

## Abstract

Prolonged sedentary behavior in modern workplaces is a recognized ergonomic risk factor linked to joint degeneration and impaired cartilage health. It has been known that Insulin-like growth factor 1 (IGF-1) plays an important role in maintaining chondrocyte activity and extracellular matrix turnover. This study presents a numerical model to investigate the influence of workplace ergonomic activities, especially repeated sit-to-stand (STS) cycles, on IGF-1 transport within cartilage. First, a musculoskeletal model was developed to quantify the knee joint reaction force and muscle loading induced by various ergonomic activities in accordance with WorkSafe Victoria’s posture-break guidelines. Then, interstitial fluid flow-induced advective transport of IGF-1 within cartilage is quantified by employing a poroelastic cartilage tissue model in conjunction with a cartilage contact model. The results demonstrate that STS activities can substantially enhance IGF-1 transport in cartilage. For instance, over a 5-hour period, an STS cycle with 10 min of sitting and 10 min of standing increased free, bound, and total IGF-1 uptake ratios by 15.8%, 9.6%, and 9.7%, respectively, compared with continuous sitting. Shorter STS cycle times yielded even greater improvements in IGF-1 uptake. Moreover, accurate representation of the contact gap proved critical, as neglecting it led to significant underestimation of solute transport.

## Introduction

Articular cartilage is a white, smooth, and highly specialized connective tissue that covers the ends of long bones such as the femur and tibia, providing a low-friction surface for load-bearing joint articulation (Smith et al. 2019; Sophia Fox et al. 2009). The mechanical properties of articular cartilage are primarily governed by its extracellular matrix (ECM), which is mainly composed of proteoglycans and collagen fibres (DiDomenico et al. 2018; Grodzinsky et al. 2000; Kempson 1980). Proteoglycans, which consist of negatively charged glycosaminoglycans (GAGs) chains, are critical in generating osmotic swelling pressure and thus contribute significantly to the cartilage’s resistance to compressive loading (Han et al. 2011; Urban et al. 1979). Meanwhile, the cross-linked collagen network provides the tensile strength required to maintain cartilage structural integrity under tensile stress (Williamson et al. 2003; Yamauchi and Mechanic 2018). This characteristic allows articular cartilage to endure repetitive cyclic loads with minimal damage, maintaining its essential role in joint function and mechanics (Buckwalter 1992).

To preserve cartilage functionality, both mechanical loading and nutrients (e.g. growth factors) are essential for maintaining the integrity and health of the ECM (Luyten et al. 1988). Among these growth factors, insulin-like growth factors (IGFs), including insulin-like growth factor 1 (IGF-1) and insulin-like growth factor 2 (IGF-2) play a central role in promoting matrix synthesis and cellular repair (Grodzinsky et al. 2000). In particular, IGF-1 has been shown to stimulate the synthesis of proteoglycans and collagen by binding to specific receptors on the surface of chondrocytes (Doré et al. 1994; Schmidt et al. 2006). The bioactivity of IGF-1 is primarily regulated by a family of six insulin-like growth factor binding proteins (IGFBPs), as well as by its interaction with cell surface receptors (Wong et al. 1999). Among them, IGFBPs 1–5 are considered in this study, as this study focuses specifically on IGF-1 regulation, while IGFBP-6 is excluded due to its preferential physiological affinity for IGF-2 rather than IGF-1 (Bach 2016). Previous studies have demonstrated that IGFBPs present within the ECM can extend the half-life of IGF-1 (Allard and Duan 2018; Anan et al. 2016). Functioning as a molecular “reservoir,” IGFBPs help modulate IGF-1 availability and control its binding to chondrocyte receptors (Paye and Forsten-Williams 2006).

IGF-1 is predominantly produced by the liver and transported via the bloodstream to peripheral joints, including the knee, and transported to the surface of the cartilage through the synovial fluid (Zhang et al. 2009, 2010). Due to the avascular nature of articular cartilage, the delivery of IGF-1 into the extracellular matrix (ECM) relies on passive diffusion and advection from the synovial fluid (DiDomenico et al. 2018; Gardiner et al. 2007; Zhang et al. 2009, 2013). In vitro studies have demonstrated that dynamic mechanical loading enhances the biosynthetic activity of chondrocytes and supports ECM maintenance more effectively than static loading (Burton-Wurster et al. 1993; Palmoski and Brandt 1984; Parkkinen et al. 1992). These effects depend on the specific loading parameters, including frequency, magnitude, and duration (Grodzinsky et al. 2000). Experimental findings by Bonassar et al. (2000) showed that dynamic loading facilitates the transport of IGF-1 from the surrounding bath into the cartilage tissue. Similarly, mathematical models developed by Gardiner et al. (2007); Zhang et al. (2008b) confirmed that dynamic loading enhances IGF-1 transport by stimulating advective mechanisms within the tissue.

However, many traditional reactive solute transport computational models simplify articular cartilage as a homogeneous, cylindrical disc with linear and isotropic material properties (Gardiner et al. 2007; Zhang et al. 2007, 2008a, b, 2009, 2010, 2013). These models typically ignore the nonlinear tension-compression behavior that varies across different cartilage zones. Since IGF-1 transport is influenced by local tissue deformation and strain (Zhang et al. 2007), it is necessary to incorporate a more realistic model that accounts for the tension–compression nonlinearity of cartilage (Miramini et al. 2017; Zhang et al. 2015). Additionally, existing models neglect the role of the contact gap and the fluid exchange between the synovial fluid and the interstitial fluid within the cartilage. Studies by Liao et al. (2019); Li et al. (2023b) have shown that the presence of a contact gap can significantly influence fluid exudation under static compression, which is important for solute transport. Therefore, it is reasonable to develop a coupled human tibial cartilage model that integrates the cartilage tissue model and the cartilage contact gap model. This would provide a more accurate simulation of IGF-1 transport under physiological joint loading conditions.

In modern society, the workplace is the primary environment where people spend extended periods of time. Prolonged static sitting has become a known risk factor for articular cartilage health, as sustained static loading can suppress cartilage matrix biosynthesis and reduce chondrocyte proliferation (Palmoski and Brandt 1984; Parkkinen et al. 1992). According to WorkSafe Australia, long-term sitting is associated with an increased risk of occupational health issues (Straker et al. 2016). Therefore, integrating different ergonomic activities (e.g., sit-to-stand) into the office routine, enabled by the use of adjustable desks, which are now common in many workplaces, may help stimulate cartilage metabolism by enhancing IGF-1 transport and activity in the joint through intermittent mechanical loading.

To further investigate IGF-1 regulation under workplace-related loading conditions, a musculoskeletal modelling approach can be adopted to quantify the mechanical loading applied to the articular cartilage surface, accounting for both external posture and muscle-induced forces (Millard et al. 2013). OpenSim, a robust open-source platform for musculoskeletal simulation, provides an effective framework for estimating joint loading, including muscle contributions and knee joint reaction forces (Li et al. 2023a, 2025b). These simulated knee contact forces can be used to inform and calibrate cartilage transport models, improving their alignment with realistic physiological conditions experienced during everyday workplace activities.

Therefore, the objective of this study is to investigate IGF-1 transport under workplace-related joint loading scenarios. A coupled mode, integrating a nonlinear cartilage tissue representation, a contact gap fluid exchange mechanism, and reactive solute transport, is developed. A series of parametric studies is conducted to examine IGF-1 concentration profiles under various workplace activity patterns.

## Materials and methods

### Study overview

This study introduces a detailed reactive transport model for IGF-1 within lateral articular cartilage based on the principles of porous media theory. This model conceptualises articular cartilage as a complex mixture of three distinct phases. These correspond to the solid phase (denoted with superscript *s*), which represents the extracellular matrix (ECM); the incompressible fluid phase (denoted with superscript *f*), accounting for the interstitial fluid permeating the cartilage; and the solute phase (denoted with superscript *w*), including both freely mobile and protein-bound IGF-1 molecules (Zhang et al. 2009). This multi-phase approach allows for a nuanced examination of the dynamics and interactions of IGF-1 in such a cartilaginous environment.

A proposed model in this study incorporates the cartilage tissue and cartilage contact gap model that describes the interaction between cartilage tissue and the contact gap to the reactive transport model for IGF-1. These two models consider the fluid flow behaviour and interactions between different regions under loading, including the flow of interstitial fluid within the cartilage tissue, the flow of synovial fluid in the cartilage contact gap, and the exchange of fluid across the contact interface (Li et al. 2023c).

To emulate the cyclical loading patterns encountered in ergonomic office settings, the study introduces different sit-to-stand (STS) exercise activity that modulate the convective and diffusive transport of IGF-1 into the cartilage. Previous studies have shown that muscle-induced forces significantly influence knee joint loading (Anan et al. 2016; Chen et al. 2020; Lin et al. 2010). Accordingly, the model integrates an OpenSim-based musculoskeletal model to provide time-resolved joint reaction forces and muscle activation profiles, thereby refining our predictions of IGF-1 delivery and enhancing the accuracy of rehabilitation outcome simulations for working individuals (Li et al. 2025a, b).

**Fig. 1:**
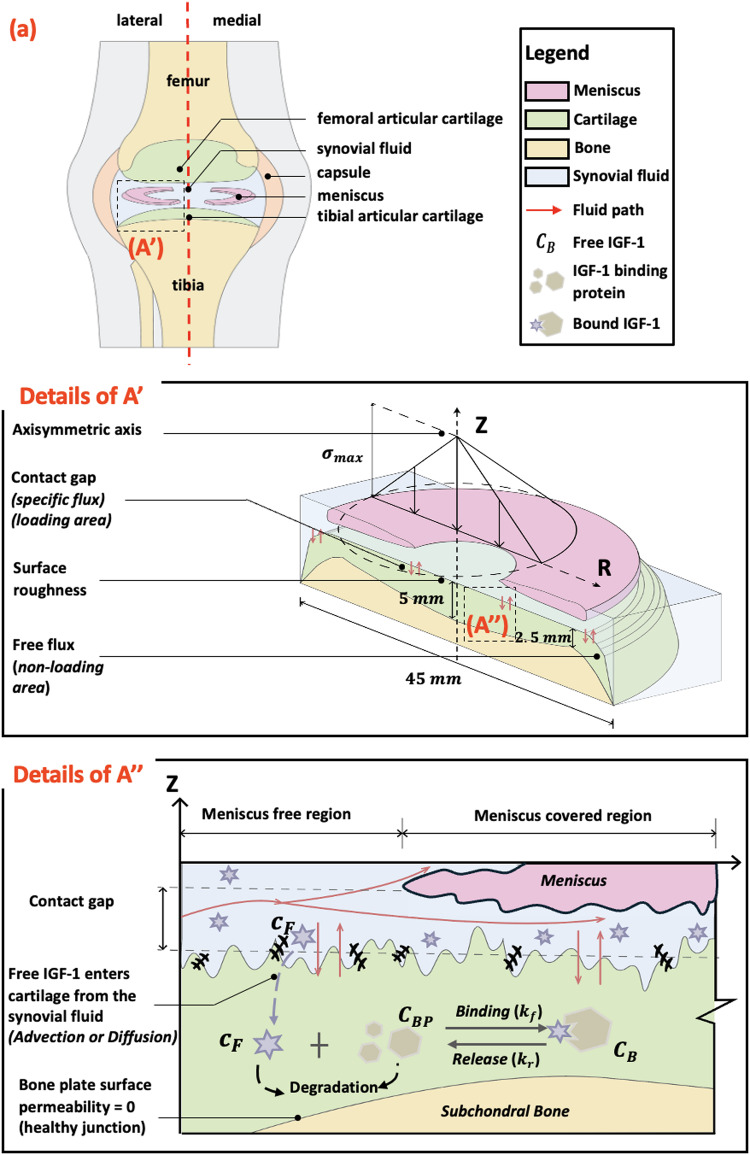
Schematic overview of the transport process of IGF-1 in cartilage tissue

**Fig. 2:**
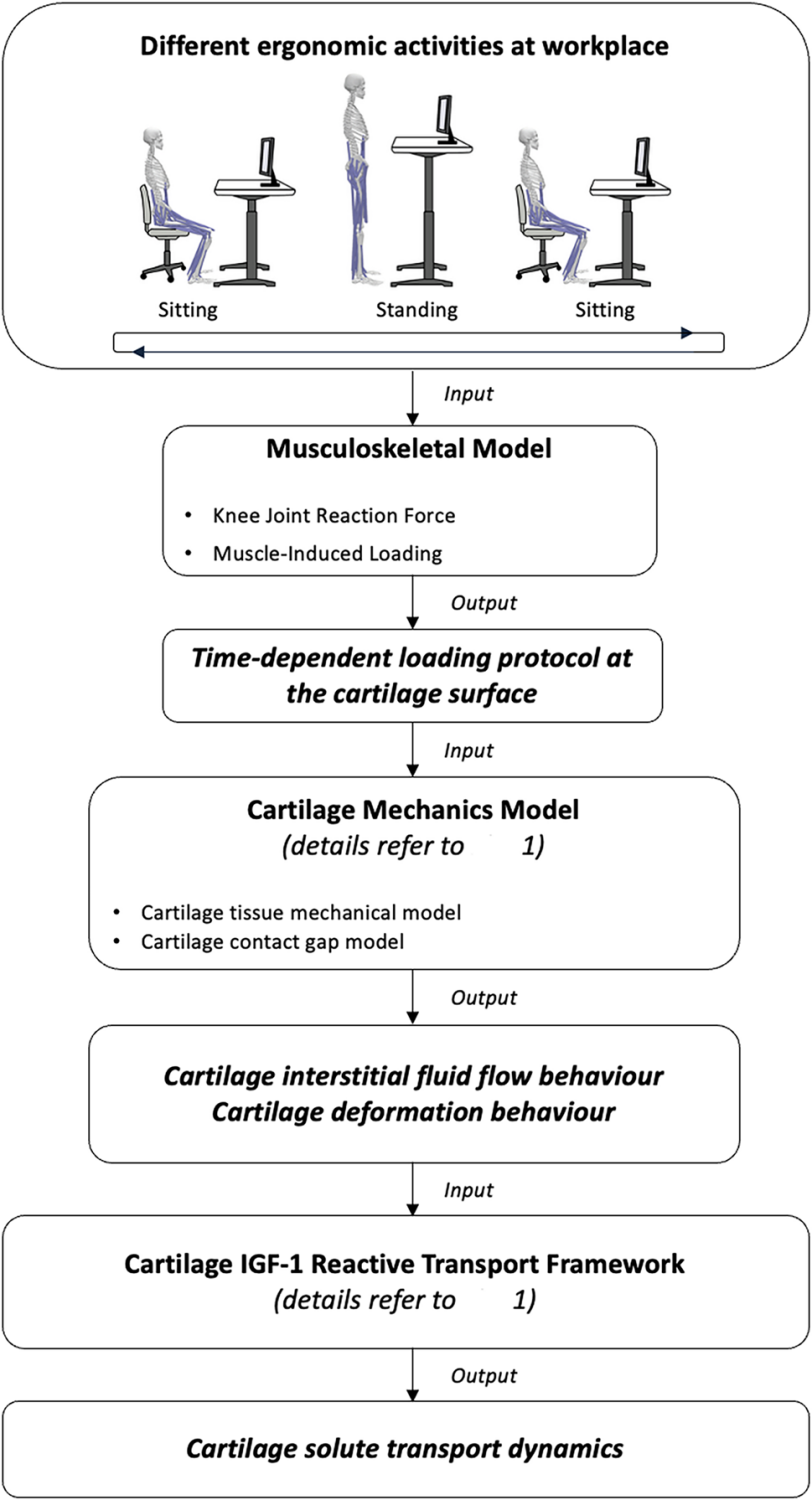
The coupled IGF-1 transport model under different workplace ergonomic activities developed in this study

The schematic overview of cartilage under dynamic loading and the potential processes of solute binding within the cartilage under dynamic loading in Fig. 1. The schematic diagram of the methodology used in this study is shown in Fig. 2.

### Assumption

While the precise mechanisms of cartilage biosynthesis are not fully understood (Zhang et al. 2008a), there is strong evidence from both in vivo and in vitro research that IGF-1 plays a crucial role in promoting the growth and development of cartilage (Martel-Pelletier et al. 1998). To streamline the modelling of IGF-1 reactive transport in the computational framework depicted in Fig. 2, the following assumptions have been adopted:

- The shape and boundary conditions of the cartilage tissue are considered to be axis-symmetric (Liao et al. 2020a; Miramini et al. 2017).
- Although IGF-1 is present in both synovial fluid and cartilage, previous experiments have substantiated that under explant culture conditions, little or nearly no IGF-1 was synthesised by chondrocytes (Luyten et al. 1988; Zhang et al. 2008a). Thus, the IGF-1 in cartilage is assumed to be mainly supplied from the synovial fluid.
- The total IGF-1 concentration ($\:{c}^{w}$) is assumed to be comprised of both free IGF-1 ($\:{c}_{F}$) and bound IGF-1 ($\:{c}_{B}$) (Zhang et al. 2009).
- The binding between IGF-2 and IGFBPs is neglected in this study.
- In this study, IGFBPs 1–5 are assumed to be immobilized within the ECM, and IGFBP turnover is neglected. Each loading cycle is assumed to have a uniform magnitude to enable direct comparison across different peak-load scenarios, and the analysis excludes loads occurring during the transition, considering only the loads experienced during static sitting and standing.

### Musculoskeletal model

Data collected from 65 healthy adults aged 19 to 73 years (Perera et al. 2024) were used to capture a broad range of knee-loading patterns relevant to workplace activities. Motion and ground reaction forces (GRFs) were recorded in the dataset for transitions from sitting to walking, encompassing 36 degrees of freedom. Using OpenSim 4.5, the model implemented the Gait2354 lower limb model to derive a time-varying knee joint loading protocol featuring 54 muscle-tendon actuators (Abdullah et al. 2024). Key muscle groups known to contribute significantly to knee loading, such as the quadriceps, hamstrings, gluteus maximus, and gastrocnemius, were included when constructing the final time-dependent loading profile (Kim et al. 2009; Lin et al. 2010; Sasaki and Neptune 2010).

Inverse kinematics was applied to compute joint angles by aligning the scaled musculoskeletal model with the recorded activity trajectories, thereby minimizing marker errors and providing a continuous description of lower-limb motion. Building on these kinematic results, GRFs from the dataset were incorporated into the simulation to represent external loading conditions. Using these two inputs, the overall knee and cartilage loading protocol was derived through static optimization and joint analysis functions in OpenSim. Specifically, static optimization estimated muscle force distributions that best balanced the measured GRFs. These outputs were then used in the joint analysis tool to compute joint reaction forces, yielding a detailed, time-dependent profile of mechanical loading on the knee cartilage (Li et al. 2023a, 2025a, b), as expressed in Eqs. (1),

1 $$\:{\overrightarrow{R}}_{i-1}={M}_{i}\left(\overrightarrow{q}\right){\overrightarrow{a}}_{i}+{\overrightarrow{F}}_{constraint}-\left(\sum\:{\overrightarrow{F}}_{external}+\sum\:{\overrightarrow{F}}_{muscles}+{\overrightarrow{R}}_{i+1}\right)$$

where $\:{M}_{i}\left(\overrightarrow{q}\right)$ is the mass matrix of the body; $\:{\overrightarrow{a}}_{i}$ represents the body acceleration vector obtained through static optimazaition function; $\:{\overrightarrow{F}}_{constraint}$, $\:{\overrightarrow{F}}_{external}$ and $\:{\overrightarrow{F}}_{muscles}$ denote the constraint, external and muscle forces, respectively; $\:{\overrightarrow{R}}_{i-1}$ and $\:{\overrightarrow{R}}_{i+1}$ are the joint reaction forces on the proximal and distal joints of $\:{i}^{th}$ component.

### Cartilage model governing equation

In the framework of the theory of porous media (Miramini et al. 2017; Zhang et al. 2007), the volume fraction of solid, fluid and solute phases can be defined respectively as follows,

2 $$\:{\varphi\:}^{s}=\frac{{V}^{s}}{V}$$

3 $$\:{\varphi\:}^{f}=\frac{{V}^{f}}{V}$$

4 $$\:{\varphi\:}^{w}=\frac{{V}^{w}}{V}$$

where $\:V$ is the volume of the mixture and $\:{V}^{s}$, $\:{V}^{f}$, $\:{V}^{w}$ are the volume of solid, fluid and solute, respectively. Since the solute phase has an extremely small volume compared to the solid and fluid phases, it can be assumed that the solute phase volume fraction equals 0. According to the definition, all volume fractions are equal to 1 (Zhang et al. 2007).

5 $$\:{\varphi\:}^{s}+{\varphi\:}^{f}\approx\:1$$

In this study, we acknowledge the framework of the previous study, where the total solute concentration ($\:{c}^{w}$) includes both free and bound solute (Zhang et al. 2008b, 2009), which can be explained as,

6 $$\:{c}_{F}+{c}_{B}={c}^{w}$$

where $\:{\boldsymbol{c}}_{\boldsymbol{F}}$, $\:{\boldsymbol{c}}_{\boldsymbol{B}}$ are the concentration of unbound and bound solute onto solid phases, respectively. Both concentrations are relative to the volume of the fluid phase.

According to Eqs. (2)–(4), the concentration of solute relative to the different phases ($\:\alpha\:$) can be determined respectively as follows,

7 $$\:{c}^{\alpha\:}=\frac{{\stackrel{-}{c}}^{\alpha\:}\:}{{\varphi\:}^{\alpha\:}}$$

where $\:{\stackrel{-}{c}}^{\alpha\:}$ is the average molar concentration in the tissue, while $\:{c}^{\alpha\:}$ is the molar concentration in the alpha phase.

#### Cartilage tissue model

The poroelastic cartilage tissue model developed by Zhang et al. characterizes the mechanical properties of cartilage by treating it as a fully saturated, deformable porous medium composed of interstitial fluid and solid phases. The model considers the aggrecan-dependent compressive modulus, collagen network-dependent tension modulus, tension-compression nonlinearity, and aggrecan-dependent permeability (Li et al. 2023c; Liao et al. 2019).

The governing equations of the cartilage tissue model are based on mass conservation and Darcy’s law (Li et al. 2023b; Liao et al. 2020b). Assuming the solid and fluid phases are incompressible, the mass conservation of both phases in the mixture is applied,

8 $$\:\nabla\:\cdot\:\left[\left(1-{\varphi\:}^{f}\right){v}^{s}+{\varphi\:}^{f}{v}^{f}\right]=0$$

The interstitial fluid flow $\:{v}_{f}$ within the cartilage tissue is governed by Darcy’s law (Liao et al. 2020b). It is assumed that the velocity of the interstitial fluid relative to the solid matrix velocity is proportional to the fluid pressure gradient (Zhang et al. 2007). Therefore, the governing Equation that illustrates the relationship between cartilage tissue deformation and interstitial fluid flow can be formulated as follows:

9 $$\:\nabla\:\cdot\:\left[{v}^{s}-{K}_{c}\nabla\:p\right]=0$$

where $\:{K}_{c}$ is the hydraulic permeability of cartilage tissue. According to the force equilibrium, Smith et al. derive the momentum conservation equation by neglecting body forces and inertia forces (Liao et al. 2020a),

10 $$\:\nabla\:\cdot\:{\sigma\:}_{t}=\nabla\:\cdot\:{(\sigma\:}_{E}^{s}-{p}_{c}I)=0$$

where $\:{\sigma\:}_{t}$ is the total stress applied, $\:{\sigma\:}_{E}^{s}$ represents the incremental effective elastic stress resulting from the deformation of the solid phase of cartilage tissue under loading, $\:{p}_{c}$ is the incremental interstitial fluid pressure, and $\:I$ is the identity tensor. And the constituent effective stress of solid phase (Cauchy stress) is represented by (Liu et al. 2023; Miramini et al. 2015),

11 $$\:{\sigma\:}_{E}^{s}=\frac{2}{{J}^{s}}{F}^{s}\cdot\:\frac{\partial\:U\left({u}^{s}\right)}{\partial\:{C}^{s}}\cdot\:{F}^{sT}$$

where $\:U\left({\mathrm{u}}^{s}\right)$ is the solid stored Helmholtz energy per unit reference volume (i.e., strain energy density), $\:{F}^{s}$ is the deformation gradient for the solid phase, $\:{\mathrm{C}}^{s}={F}^{sT}\times\:{F}^{s}$ is the right Cauchy-Green deformation tensor for the solid phase, and $\:{J}^{s}={det}\left({F}^{s}\right)$ is the solid phase volume change ratio (Liu et al. 2023; Miramini et al. 2015).

Aggrecan content-dependent compressive modulus and the tension-compression nonlinearity are incorporated into the model. Based on the experiments on human cartilage by Treppo et al. (2000), the relationship between cartilage compressive stiffness and aggrecan content is characterized by,

12 $$\:{H}_{A}={\alpha\:}_{1}{\varphi\:}_{G}+{\alpha\:}_{2}{\varphi\:}_{G}^{2}$$

where $\:{H}_{A}$ is the aggregate modulus of cartilage, $\:{\varphi\:}_{G}$ represents the “actual” aggrecan content, while the empirical constants $\:{\alpha\:}_{1}$ and $\:{\alpha\:}_{2}$ are set at 0.01 MPa and 0.075 MPa, respectively (Treppo et al. 2000). The “actual” aggrecan content $\:{\varphi\:}_{G}$ varies with cartilage volumetric deformation, which can be expressed as, wo

13 $$\:{\varphi\:}_{G}=\frac{{\varphi\:}_{G0}}{{J}^{s}-\alpha\:}$$

where $\:{\varphi\:}_{G0}$ is the depth-dependent initial aggrecan content under no external loading condition. Based on the mapped profile through magnetic resonance imaging (MRI) (Xia et al. 2008), $\:{\varphi\:}_{G0}$ can be obtained by linear interpolation between $\:{\varphi\:}_{G0}\left(z=0mm\right)=30mg/ml$ and $\:{\varphi\:}_{G0}\left(z=5mm\right)=120mg/ml$ (Li et al. 2023c; Zhang et al. 2015). $\:\alpha\:$ is the depth-dependent collagen volume fraction of cartilage, taken as 45%, 30% and 25% for the superficial zone, middle zone and deep zone, respectively (Liao et al. 2020b; Miramini et al. 2017).

The elastic compressive modulus of cartilage $\:{E}_{c}$ can be obtained from the aggregate modules (Smith et al. 2019) by using the Equation below:

14 $$\:{E}_{c}=3{H}_{A}(1-2\upsilon\:)$$

where $\:\upsilon\:$ is the aggrecan effective Poisson’s ratio (Miramini et al. 2017). The collagen network in cartilage contributes tensile stiffness to the matrix. Notably, the orientation and concentration of collagen fibrils change depending on the depth within the cartilage, resulting in a tensile modulus that varies by depth and orientation. The tensile modulus values applied in this research are detailed in Table 1 (Liao et al. 2019; Miramini et al. 2017).

**Table 1:** Tensile and shear modulus used in this study (Liao et al. 2019)

Zones	Tensile Modulus (MPa)		Shear Modulus (MPa)
	Horizontal	Vertical	
Superficial Zone	100	25	3
Middle Zone	30	10	3
Deep Zone	10	15	2

The negatively charged characteristics of aggrecan impact the flow resistance within cartilage. Experimental findings indicate an inverse relationship between aggrecan content and hydraulic permeability represented by Eqs. (15),

15 $$\:{K}_{c}=\frac{n\cdot\:{\left({\varphi\:}_{G}\right)}^{m}}{\eta\:}$$

where $\:n$ and $\:m$ are empirical constants taken as $\:5.4\times\:{10}^{-22}{m}^{2}$ and − 2.37, respectively. The viscosity of water is denoted as $\:\eta\:$, is considered to be 0.0007 Pa$\:\cdot\:$s at 37 °C (Miramini et al. 2017).

#### Contact gap model

The contact gap model developed by Liao et al. explores the flow of synovial fluid within the contact gap and the deformation of surface roughness, and it is integrated with the cartilage tissue model through the continuity of pressure and normal flux at boundary conditions (Liao et al. 2020a, b). This model includes synovial fluid in the gap and the porous spaces created by surface asperities upon initial contact. Governed by the law of mass conservation and Darcy’s law, the model uses a constitutive equation in exponential form to describe the local deformation of asperities during contact. The flow of synovial fluid in the contact gap is modelled as the form of Darcy’s law (Li et al. 2023c) as shown in Eqs. (16),

16 $$\:{v}_{d}^{g}=-{K}_{g}\cdot\:\nabla\:{p}_{g}$$

where $\:{v}_{d}^{g}$ is the Darcy velocity of synovial fluid in the contact gap, $\:{K}_{g}$ is the gap permeability and $\:{p}_{g}$ is the fluid pressure in the gap. The permeability of the contact gap is mainly determined by the gap height and the surface roughness, which are indicators of the lateral fluid flow capability of the contact gap. The permeability values were numerically assessed using the developed computational fluid dynamics model, considering the experimentally measured surface roughness of the bovine medial tibia. Further details are available in previous studies (Liao et al. 2020b).

The principle of mass conservation governs the fluid exchange between the cartilage tissue and the contact gap,

17 $$\:\frac{\partial\:{\epsilon\:}_{v}^{g}}{\partial\:t}+\nabla\:\cdot\:{v}_{d}^{g}=s$$

where $\:\frac{\partial\:{\epsilon\:}_{v}^{g}}{\partial\:t}$ is the change rate of the gap volumetric strain, $\:s$ is the change rate of the fluid exchange per unit volume. The volumetric strain of the contact gap $\:{\epsilon\:}_{v}^{g}$ is connected to the gap height $\:h$, and the relationship between gap closure and contact stress is described using an exponential equation derived from experimental data on human cartilage (Deneweth et al. 2013; Robinson et al. 2016).

18 $$\:{\sigma\:}_{t}={\sigma\:}_{c}-{p}_{g}$$

19 $$\:h={h}_{0}{e}^{{\sigma\:}_{c}/\beta\:}={h}_{0}{e}^{({\sigma\:}_{t}+{p}_{g})/\beta\:}$$

where $\:{\sigma\:}_{c}$ is the contact stress of the asperity, $\:{h}_{0}$ is taken as $\:9\mu\:m$ representing the highest undeformed gap height when the initial loading is applied. $\:\beta\:$ represents the stiffness of the cartilage asperities, which is set at 20% of the aggregate modulus (Graindorge et al. 2005). Detailed descriptions of the contact gap model can be found in previous work (Liao et al. 2020a).

#### Reactive transport model of IGF-1

The reactive transport model of IGF-1 was developed by Zhang et al. within the context of porous media theory modelling configurations and boundary conditions (Zhang et al. 2008a, b), and the model has been validated against experimental results (Bonassar et al. 2000). In the current study, we have expanded this model by integrating the coupled cartilage tissue and contact gap model with the reactive transport model. The binding complex is proportional to the concentrations of free IGF-1 and IGF Binding Proteins (IGFBPs) (Giustina et al. 2008). The reversible chemical reaction between IGF-1 and IGFBPs can be described by:

20 $${\mathrm{IGF}} - 1 + {\mathrm{IGFBPs}}\begin{array}{c}{{k_f}} \\ {\overleftrightarrow {{k_r}}} \\ \end{array} {\mathrm{IGF}} - 1/{\mathrm{IGFBPs~Complex}}$$

where $\:{k}_{f}$ and $\:{k}_{r}$ represent the reaction rate constant for the association reaction and dissociation reaction, which have been estimated from the previous experiments (Cassino 2002). The chemical reaction in Eq. (20) can be described mathematically using the law of mass action (Gardiner et al. 2007; Zhang et al. 2007, 2008a, b, 2010), which assumes that the concentration of bound IGF-1 ($\:{c}_{B}$) is directly proportional to the concentration of the reactants (e.g. the free/unbound IGF-1 ($\:{c}_{F}$) and IGFBPs ($\:{c}_{BP}$), and the reaction rate is directly proportional to the product of the molar concentrations of the involved molecules. (i.e., $\:{\stackrel{-}{c}}_{F}$ for free IGF-1, $\:{\stackrel{-}{c}}_{B}$ for bound IGF-1 and $\:{\stackrel{-}{c}}_{BP}$ for IGFBPs). Using the law of mass action (Zhang et al. 2009, 2010, 2013), the reversible chemical reaction in Eq. (20) can be expressed as:

21 $$\begin{aligned}\:{\varphi\:}^{f}\frac{\partial\:{\stackrel{-}{c}}_{F}}{\partial\:t}&=-\nabla\:\cdot\:\left(-{\varphi\:}^{f}D\nabla\:{\stackrel{-}{c}}_{F}+{\varphi\:}^{f}{v}^{f}{\stackrel{-}{c}}_{F}\right)-{\varphi\:}^{f}\left(1-{\varphi\:}^{f}\right){k}_{f}{\stackrel{-}{c}}_{F}{\stackrel{-}{c}}_{BP}\\&+\left(1-{\varphi\:}^{f}\right){k}_{r}{\stackrel{-}{c}}_{B}-{{\varphi\:}^{f}k}_{d}{\stackrel{-}{c}}_{F}\end{aligned}$$

22 $$\:\frac{\partial\:{\stackrel{-}{c}}_{B}}{\partial\:t}=-\nabla\:\cdot\:\left({v}^{s}{\stackrel{-}{c}}_{B}\right)+{\varphi\:}^{f}{k}_{f}{\stackrel{-}{c}}_{F}{\stackrel{-}{c}}_{BP}-{k}_{r}{\stackrel{-}{c}}_{B}$$

23 $$\:\frac{\partial\:{\stackrel{-}{c}}_{BP}}{\partial\:t}=-{\varphi\:}^{f}{k}_{f}{\stackrel{-}{c}}_{F}{\stackrel{-}{c}}_{BP}+{k}_{r}{\stackrel{-}{c}}_{B}$$

where $\:{\varphi\:}^{f}$ is the volume fraction of the fluid phase, $\:{v}^{f}$ is the fluid phase velocity, which can be obtained by coupling the cartilage tissue and contact gap models and $\:{v}^{s}$ is the solid phase velocity. $\:{\stackrel{-}{c}}_{F}$, $\:{\stackrel{-}{c}}_{BP}$ and $\:{\stackrel{-}{c}}_{B}$ are volume-based average concentrations for free IGF-1, IGFBPs and bound IGF-1, respectively. IGF-1 can be transported from synovial fluid into the cartilage by diffusion and advection, as described by Eq. (21), $\:D$ is the diffusion coefficient of IGF-1. $\:{k}_{d}$ is the degradation constant for free IGF-1. It is assumed that most IGF-1 is sourced from synovial fluid outside the cartilage (Zhang et al. 2008a, b).

### Modelling configurations and boundary conditions

#### Model geometry

The geometry of the lateral tibial cartilage model of this study is developed based on MRI measurements developed by Goodwin (Goodwin et al. 2004). The thickness of cartilage in the symmetry axis is 5 mm (Li et al. 2023b, c), and the detailed geometry shape is shown in Figs. 2 and 4.

#### Loading protocol

To streamline the computational framework, all time-dependent loads are applied purely in the axial direction. The report assumes the load to be equally distributed between lateral and medial compartments of the knee. To isolate the effects of distinct workplace activities on IGF-1 transport, each simulation scenario assumes a uniform body mass of 75 kg. Free diffusion cases serve as reference conditions, devoid of any mechanical loading, providing a baseline distribution of IGF-1 under quiescent conditions.

**Fig. 3:**
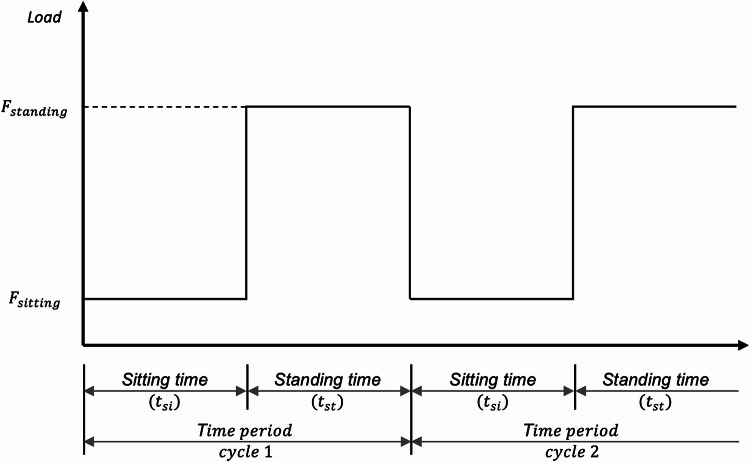
Loading protocols simulating workplace ergonomic activities used in this study. Each loading cycle consists of sitting time ($\:{t}_{si}$), standing time ($\:{t}_{st}$) (i.e. time period $\:{T=\:{t}_{si}+\:t}_{st}$), standing load ($\:{F}_{standing}$) and sitting load ($\:{F}_{sitting}$)

According to WorkSafe Australia (Straker et al. 2016), employees are recommended to take a break after 30 min of continuous sitting at work to help minimize potential health risks. In line with this guidance, cyclic loading protocols in this study begin with a 30-minute sitting period. Figure 3 illustrates the loading protocols designed to simulate workplace ergonomics. To assess the influence of mechanical activity on IGF-1 diffusion and advection, different intensities of sit-to-stand (STS) movements were applied under varying loading conditions, as summarized in Table 2.

**Table 2:** Effect of different workplace ergonomic activities on IGF-1 transport in cartilage

Cases	Activitiy	Loading time interval
Control Case (free diffusion)	N/A	N/A
Case 1	Sitting for 5 h	N/A
Case 2	Sit to Stand for 5 h	$\:{t}_{si}={t}_{st}=30min$
Case 3	Sit to Stand for 5 h	$\:{t}_{si}={t}_{st}=20min$
Case 4	Sit to Stand for 5 h	$\:{t}_{si}={t}_{st}=15min$
Case 5	Sit to Stand for 5 h	$\:{t}_{si}={t}_{st}=10min$

#### Boundary conditions

**Fig. 4:**
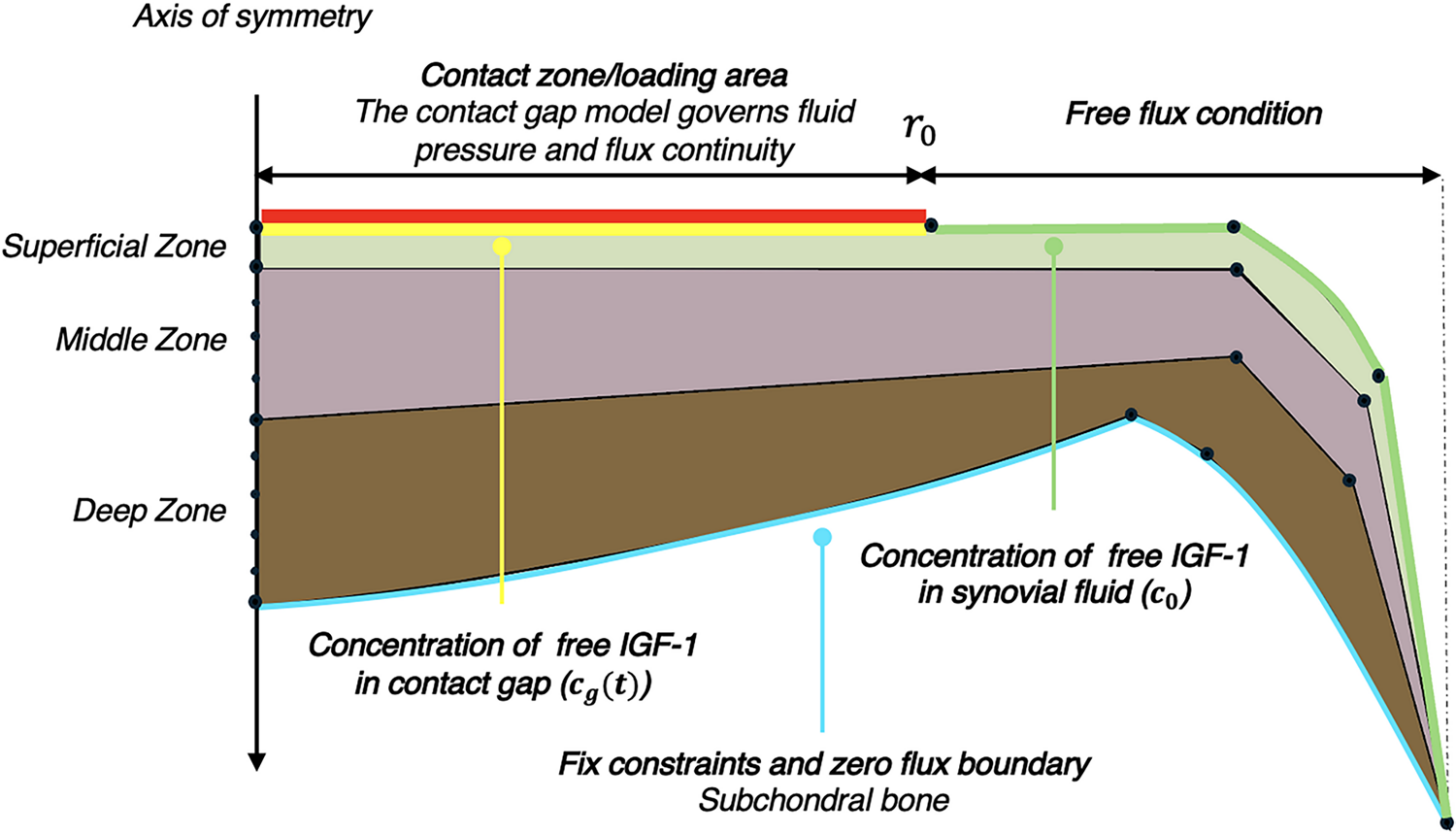
Boundary conditions used in this study

Several boundary conditions are applied to connect these three sets of governing equations, as summarised in Fig. 4.

- The continuity of normal flux condition is applied at the contact interface, dominated by mass conservation in the contact gap model (Li et al. 2023b, c; Liao et al. 2019, 2020a).
- Free flux condition is applied at the perimeter edge of cartilage tissue in the non-contact zone (Gbehe et al. 2016; Liao et al. 2020a, b; Nabhani et al. 2013; Zhang et al. 2015).
- Zero flux boundary is assumed between cartilage and subchondral bone for a healthy knee joint.
- The flux continuity condition ensures that both fluid pressure and normal fluid velocity are consistent at the interface between the upper surface of the cartilage tissue and the contact gap (Liao et al. 2020a).
- Due to the axis-symmetric assumption, all velocities are zero at $\:r=0$ (Zhang et al. 2007).Along the upper boundary surface of the cartilage tissue, non-loading cartilage regions are assumed to remain continuously exposed to bulk synovial fluid and are prescribed a steady concentration ($\:{c}_{0}$).
- At the loading area, the mass of IGF-1 is assumed to be constant and the IGF-1 concentration is assumed to be governed by a contact gap synovial reservoir that is refreshed to the bulk synovial concentration at each ergonomic posture break, where $\:\stackrel{-}{h}\left(t\right)$ is the average time-dependent contact gap height.

24 $$\:{c}_{g}\left(t\right)=\left\{\begin{array}{c}\frac{{c}_{0}*{h}_{0}}{\stackrel{-}{h}\left(t\right)},during\:compressed\:static\:contact\\\:{c}_{0\:},\:\:\:\:\:\:\:\:\:\:\:\:immediately\:after\:posture\:break\end{array}\right.$$

#### Initial conditions

The numerical modelling of the contact gap model begins with the initial contact of cartilage surface asperities. Surface asperities have yet to deform at this moment ($\:t=0$), and the highest asperity height $\:{h}_{0}$ is defined as the initial gap height. There is no effective elastic stress before the initial contact, so the total applied stress is fully counteracted by synovial fluid pressure in the gap $\:{p}_{g}$.

25 $$\:h\left(t=0\right)={h}_{0},\:\:{\sigma\:}_{t}\left(t=0\right)=-{p}_{g}$$

For the reactive transport model, it is assumed that the initial conditions are constant throughout the cartilage tissue; Therefore,

26 $$\:{c}_{F}\left(t=0\right)=0,\:\:{c}_{B}\left(t=0\right)=0,\:\:{c}_{BP}\left(t=0\right)={c}_{bp0}$$

Equation (26) describes the initial conditions for free, bound IGF-1 and IGF-1 binding protein concentration within the cartilage tissue.

### Numerical considerations

The time-dependent cartilage surface loading protocol was generated using the OpenSim 4.4, a musculoskeletal movement simulation software. Numerical solutions for the coupled cartilage mechanics and IGF-1 transport equations were obtained in COMSOL Multiphysics 6.2 (COMSOL, Inc.). A two-dimensional, axisymmetric geometry in the radial direction was discretized with 3,532 triangular finite elements. A relative tolerance of 0.01 and an absolute tolerance of 0.001 were set for all numerical computations. The solute transport parameters used throughout this study are summarised in Table 3.

**Table 3:** Parameters used throughout this study

Parameters	Values	References
Diffusion coefficient of IGF-1 $\:\left(\boldsymbol{D}\right)$	$\:\left(2-4\right)\times\:{10}^{-7}\:\mathrm{c}{\mathrm{m}}^{2}/\mathrm{s}$	Garcia et al. (2003)
Fluid phase volumetric fraction $\:\left({\boldsymbol{\varnothing\:}}_{\boldsymbol{f}}\right)$	0.8	Bonassar et al. (2000) and Mow et al. (1990)
IGF-1 concentration in healthy human synovial fluid $\:\left({\boldsymbol{c}}_{0}\right)$	$\:0.066\:\mathrm{n}\mathrm{M}$	Schneiderman et al. (1995)
Initial fixed IGFBPs 1–5 concentration in human cartilage $\:\left({\boldsymbol{c}}_{\boldsymbol{B}\boldsymbol{P}0}\right)$	$\:3-10\:\mathrm{n}\mathrm{M}$	Eviatar et al. (2003)
Association rate constant $\:\left({\boldsymbol{k}}_{\boldsymbol{f}}\right)$	$\:9.1\times\:{10}^{5}\:{\mathrm{M}}^{-1}{\mathrm{s}}^{-1}$	Wong et al. (1999)
Dissociation rate constant $\:\left({\boldsymbol{k}}_{\boldsymbol{r}}\right)$	$\:5.3\times\:{10}^{-5}\:{\mathrm{s}}^{-1}$	Wong et al. (1999)
Free IGF-1 degradation rate constant $\:\left({k}_{d}\right)$	$\:5.8\times\:{10}^{-4}\:{s}^{-1}$	Zhang et al. (2013)

## Results and discussion

In this study, we employ uptake ratios comprising the total solute uptake ratio ($\:{R}_{u}$), free or unbound solute uptake ratio ($\:{R}_{f}$) and bound solute uptake ratio ($\:{R}_{b}$). The equations of these parameters refer to the previous study (Zhang et al. 2007), These parameters indicate the ratio of solute concentration within the cartilage tissue compared to the concentration in the synovial fluid. It is assumed that the total solute uptake ratio is the sum of the free solute uptake ratio and the bound solute uptake ratio,

27 $$\:{R}_{u}={R}_{f}+{R}_{b}=\frac{{c}_{F}}{{c}_{0}}+\frac{{c}_{B}}{{c}_{0}}$$

where $\:{c}_{0}$ is the solute concentration in the synovial fluid. Since solute levels within the cartilage vary radially, the average solute uptake ratio is applied to provide a representative measure of overall solute concentration.

28 $$\:{\stackrel{-}{R}}_{u}={\stackrel{-}{R}}_{f}+{\stackrel{-}{R}}_{b}$$

The average free solute uptake ratio $\:{\stackrel{-}{R}}_{f}$ and average bound solute uptake ratio $\:{\stackrel{-}{R}}_{b}$ can be obtained by the software’s subdomain integration.

### Knee contact force

**Fig. 5:**
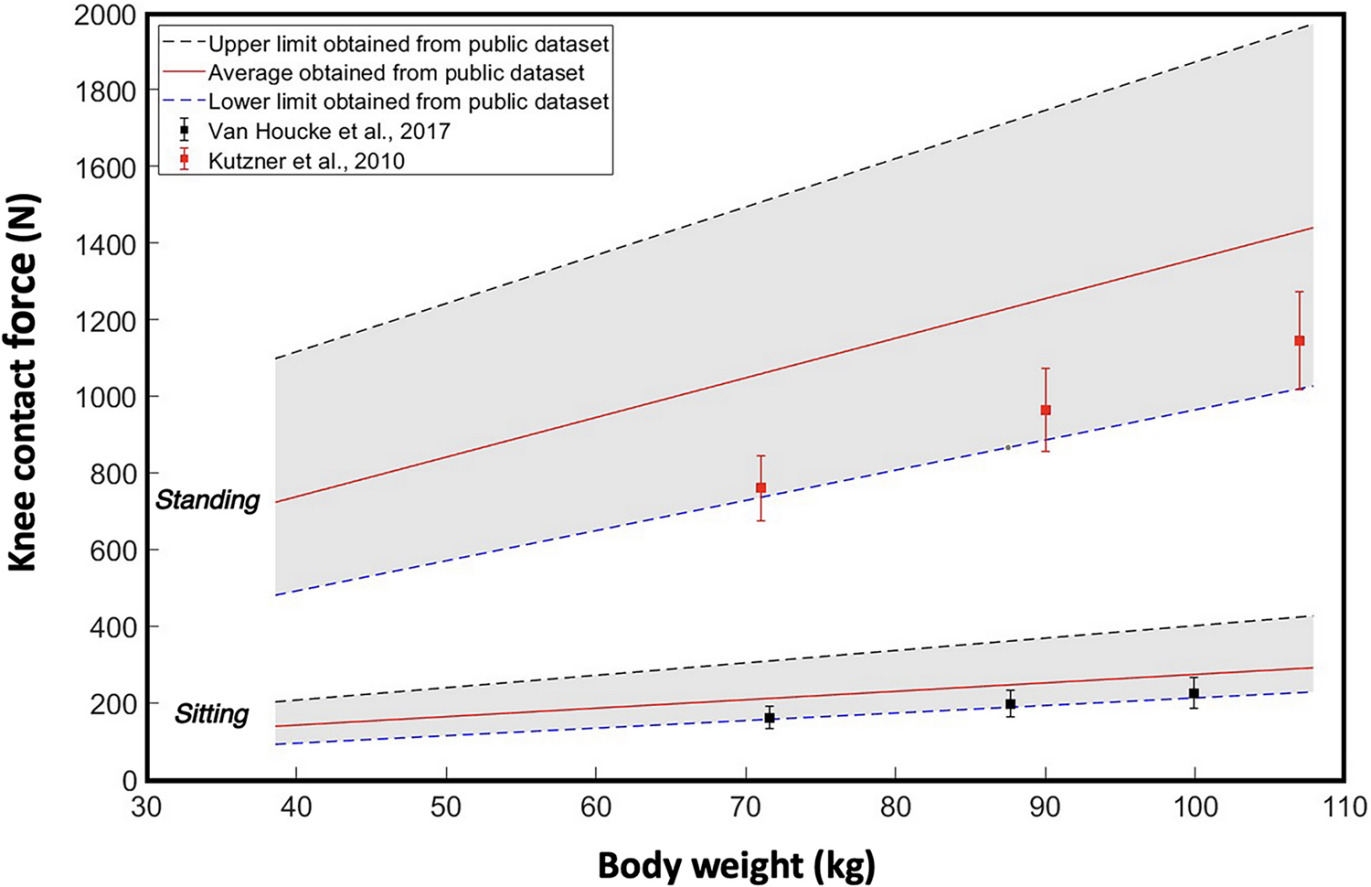
Relationship between knee contact force and body weight force during standing and sitting

Figure 5 presents the net knee contact force predicted by our musculoskeletal model across a range of subject body masses under two postural conditions: standing (top band) and sitting (bottom band). The shaded regions, defined by the dashed grey line (upper bound) and dashed blue line (lower bound), represent the model’s predicted range, while the solid red line indicates the model’s average estimated force for each condition.

Superimposed are the data points from Van Houcke et al. (Van Houcke et al. 2017) and Kutzner et al. (Kutzner et al. 2010), each with its error bar. This close alignment confirms that our model accurately captures the mass‑dependent trend of knee loading. The fact that the points cluster nearer the lower bound than the mean is expected, since our model includes most of the muscle-induced forces on the knee joint (Anan et al. 2016; Kim et al. 2009; Sasaki and Neptune 2010), leading to a modest overprediction (Trepczynski et al. 2018).

Because all validated in vivo measurements fall within the predicted range, and to align with typical physiological loading magnitudes, we will adopt the model’s average prediction (solid red line) as the input loading condition for subsequent time-dependent simulations of cartilage surface contact mechanics.

### Cartilage mechanics and IGF-1 transport

While the primary case studies in this work focused on workplace ergonomic activities, additional model validation was conducted under static standing loading against established experimental data. The cartilage mechanics model simulated peak contact strains of 9%, 14%, and 22% at 50 s, 300 s, and steady state, respectively. These results are consistent with MRI measurements reporting strains of 9% (± 3%) at 50 s and 13% (± 3%) at 300 s (Hosseini et al. 2010), as well as with the classic study by Barker and Seedhom (2001), who reported an average peak strain of 25% (± 3%) in lateral cartilage. This benchmarking exercise supports the fidelity of the cartilage mechanics model, including the incorporation of the contact gap. Furthermore, the IGF-1 transport framework has been independently validated against experimental measurements of IGF-1 diffusion in cartilage (Bhakta et al. 2000), as reported in previous work (Zhang et al. 2007).

#### Effects of the contact gap

None of the existing studies on IGF-1 transport behaviour in articular cartilage has considered the contact gap, which is present in detail A’’ of Fig. 1. The traditional transport model does not account for the gap between the two contact cartilages and the exchange of fluid, treating the contact interface as zero flux boundary, thereby leading to the inaccurate estimation of the interstitial fluid pressure and underestimating the fluid flux. Because the pressure difference between the contact gap and the cartilage tissue could significantly affect the advection behaviour in free IGF-1 transport by affecting the flow of interstitial fluid (Liao et al. 2019), it is therefore crucial to include the interaction between the contact gap and cartilage to investigate the IGF-1 transport behaviour in cartilage.

The objective of this section is to evaluate how incorporating the contact gap influences solute transport within cartilage tissue. This is examined by comparing numerical predictions of IGF-1 uptake with and without the contact gap under workplace ergonomic activity at $\:{t}_{si}={t}_{st}=30min$ (Case 2).

**Fig. 6:**
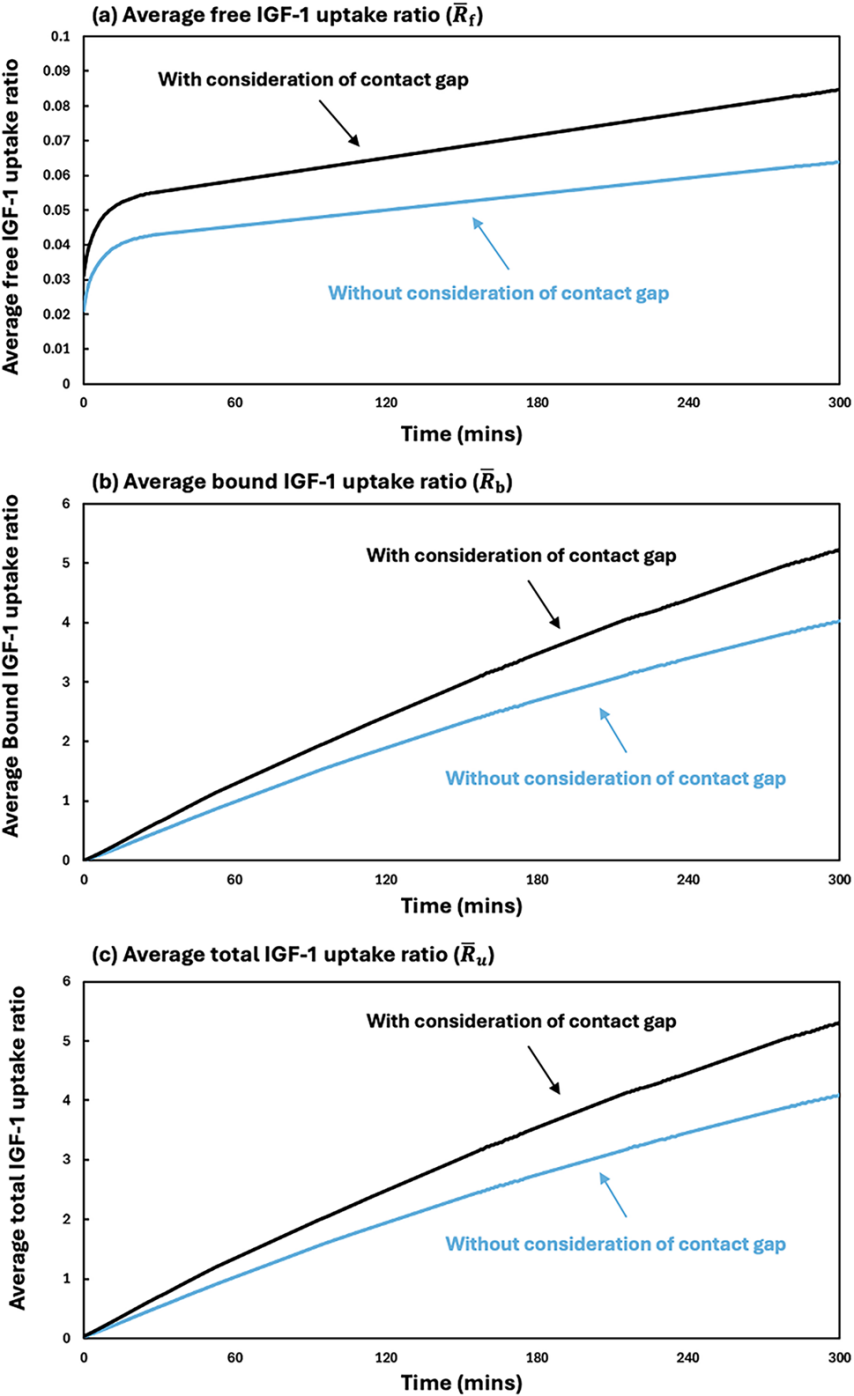
IGF-1 uptake ratios in cartilage under Case 2 ($\:{t}_{si}={t}_{st}=30min$), with and without consideration of the cartilage contact gap. (**a**) Free IGF-1 uptake ratio ($\:{\stackrel{-}{R}}_{f}$); (**b**) Bound IGF-1 uptake ratio ($\:{\stackrel{-}{R}}_{b}$); and (**c**) Total solute uptake ratio ($\:{\stackrel{-}{R}}_{u}$)

The average IGF-1 uptake ratios are consistently higher when the contact gap is considered compared with the model that excludes it. As Fig. 6. Shown, this enhancement arises because the contact gap permits flux continuity across the contact zone, leading to greater increases in free, bound, and total IGF-1 uptake. Moreover, the difference in free IGF-1 uptake develops more rapidly than in the bound or total uptake ratios. This is primarily because free IGF-1 transport is strongly influenced by interstitial fluid velocity, which is more sensitive to the presence of the contact gap. After 5 h of workplace ergonomic activity, the model incorporating the contact gap predicted a free IGF-1 uptake ratio of 0.085, compared with only 0.063 without the contact gap. Similarly, the bound and total uptake ratios reached 5.23 and 5.32 with the contact gap, whereas the corresponding values without the contact gap were lower, at 4.03 and 4.11, respectively.

Therefore, from the data shown in no flux at the contact zone. As a result, the normal fluid outflow at the contact interface is restricted, and fluid can only exude through the side surface. In the absence of the contact gap, solute transport into the cartilage tissue at the contact interface is solely governed by diffusion. However, when considering the contact gap, a certain amount of fluid is able to flow into the contact interface through the top surface of the cartilage, where it becomes confined within the interconnected spaces formed by the surface roughness (Liao et al. 2019, 2020b). Additionally, in models that do not consider the contact gap, potential time-dependent variations in free IGF-1 concentration at the contact interface are neglected. This leads to a notable contribution of fluid in supporting the load at the early contact stage, thereby enhancing the advection of solute at the contact interface due to the high-pressure gradient.

In the following sections, this refined contact gap model will be used to evaluate how different ergonomic activities influence solute transport behaviour, providing a more comprehensive understanding of the IGF-1 delivery in articular cartilage.

#### Effect of different ergonomic activities

Previous research has demonstrated that cyclic loading significantly enhances solute transport through articular cartilage, as supported by both experimental and numerical studies (Bonassar et al. 2000; Smith et al. 2019; Zhang et al. 2007). And these studies also revealed that there is a correlation between the loading time interval and the enhancement effect of solute transport. In a workplace context, this interval reflects how often individuals change posture, such as alternating between sitting and standing. It is recommended that individuals take a break or change posture after 30 min of continuous sitting, according to WorkSafe Australia (Straker et al. 2016). Based on this guideline, the current study compares the average free solute uptake ratio ($\:{\stackrel{-}{R}}_{f}$), average bound solute uptake ratio ($\:{\stackrel{-}{R}}_{b}$), and average total solute uptake ratio ($\:{\stackrel{-}{R}}_{u}$) of different cyclic STS loading periods, ranging from 30-minute to 10-minute intervals, as well as under static sitting conditions.

**Fig. 7:**
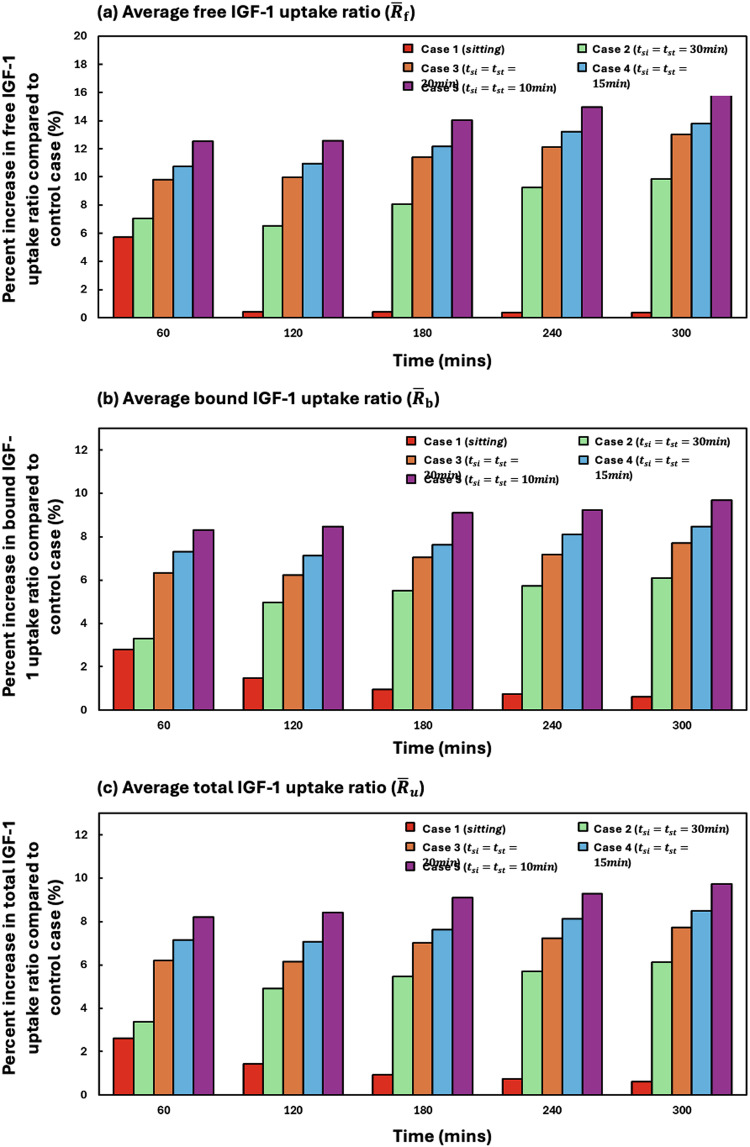
Percent increase in IGF-1 uptake ratio under different ergonomic activities compared to the control case (free diffusion). (**a**) Free IGF-1 uptake ratio ($\:{\stackrel{-}{R}}_{f}$); (**b**) Bound IGF-1 uptake ratio ($\:{\stackrel{-}{R}}_{b}$); and (**c**) Total solute uptake ratio ($\:{\stackrel{-}{R}}_{u}$)

Figure 7 depicts the relative percent increase in the average free, bound, and average total solute uptake ratio for various workplace activities in comparison to the control case of free diffusion with consideration of the contact gap across a 300-minute period. The model predicts that more frequent STS transitions result in greater solute uptake enhancement. The greatest improvements were observed in the case of $\:{t}_{si}={t}_{st}=10min$ (Case 5), with increases of approximately 15.8%, 9.6%, and 9.7% in free​, bound and total uptake ratio respectively, after 5 h. Notably, at $\:{t}_{si}={t}_{st}=30min$ (Case 2), which aligns with occupational health recommendations, demonstrated a notable improvement compared to static sitting. After five hours of simulated workplace activity, this condition resulted in approximately 9.8%, 6.0%, and 6.1% increases in free, bound, and total IGF-1 uptake ratios, respectively. In contrast, the sitting-only condition (Case 1) produced the lowest uptake ratios across all metrics, with values of approximately 0.5%, essentially indistinguishable from the free-diffusion baseline. This is reasonable, as a sustained static load does not generate advective transport after the initial load, cartilage transport reverts to being diffusion dominated, resulting in uptake values similar to the free diffusion case (Gardiner et al. 2007). These findings suggest that relatively light and feasible workplace interventions, such as standing up every 30 min, can meaningfully enhance IGF-1 transport through cartilage. This highlights the therapeutic potential of simple workplace activities in significantly mitigating the negative impact of prolonged sedentary behavior on cartilage health.

Among the three solute uptake ratios, the free (unbound) IGF-1 uptake ratio is particularly noteworthy, as the free IGF-1 can enhance the production of protein and glycan through binding to the cell surface receptor and prompting the intracellular signalling pathways of chondrocytes (Zhang et al. 2008a). The relative increase in free IGF-1 uptake for various ergonomic activities compared to static sitting ranges from 3% to 8% in the first hours. This enhancement grows to approximately 6% to 12% after the second hour, and eventually reaches about 9% to 14% after five hours of activity. This progressive improvement is primarily attributed to the continued development of interstitial fluid velocity within the cartilage induced by repeated STS movements. In contrast, under static sitting, the fluid velocity tends to diminish over time as the tissue strain approaches steady state (Li et al. 2023b), thereby reducing solute transport efficiency. As shown in Fig. 7a the percentage increase in free IGF-1 uptake for the static sitting condition begins to plateau or even decline after one hour, while the STS cases continue to show an upward trend. The results demonstrate that it increases steadily with more frequent STS interventions, indicating that mechanical loading enhances not only solute delivery but also the bioavailable fraction responsible for cellular activity.

For the bound and total IGF-1 uptake ratios, similar trends can be observed in Fig. 7b and c. Solute uptake increases with loading time interval, with a shorter loading time interval promoting greater retention of IGF-1 within the cartilage matrix. The improvement compared to static sitting becomes more apparent after the first hour of activity. As with the free IGF-1 uptake ratio, the uptake ratios in STS cases continue to rise after the first hour, whereas in the sitting condition, they tend to decrease and stabilize, indicating a steady-state condition.

Additionally, Fig. 7 shows that for all STS cases, the rate of improvement diminishes over longer durations. For example, in Case 2 ($\:{t}_{si}={t}_{st}=30min$), the increase in free IGF-1 uptake drops from approximately 3% between 1 and 2 h to less than 1% between 4 and 5 h. A similar pattern is seen in bound and total uptake ratios, with their gains decreasing from around 2% to 1% over the same periods. This diminishing increase over time aligns with findings from previous studies (Gardiner et al. 2007), where the initial mechanical stimulus induces the largest enhancement in solute transport and then plateaus with prolonged activity.

Overall, the model provides valuable insight into the physiological benefits of simple ergonomic posture-change strategies in the workplace. Using adjustable desks in modern offices can significantly enhance solute transport for free, bound, and total IGF-1 compared to static sitting. This effect is especially prominent during longer durations of activity. For individuals engaged in full-time sedentary work (e.g., over 7 h per day), incorporating regular ergonomic STS activities can meaningfully increase IGF-1 concentration in cartilage, potentially leading to improved cartilage health and better knee joint function over time.

### Limitation

It is important to note that there are certain limitations in the present study. Firstly, it should be mentioned that the amount of IGFBPs in cartilage consists of free IGFBPs and those fixed in the ECM. This study mainly focuses on the role of fixed IGFBPs on IGR-1 transport, and the effect of free IGFBPs should be further investigated in future studies. However, instead of binding to IGFBPs, IGF-1 can also bind to the cell surface receptor to increase the production of cartilage matrix. Furthermore, the degradation half-lives of IGFBPs and IGF-1 complexes were not taken into account. This study should be viewed as the first step of investigation and future studies should consider the interaction between IGF-1 and cell surface receptors and the production of aggrecans, as well as the half-lives of IGFBPs and IGF-1 complexes.

Secondly, to simplify the problem, the geometry of the lateral cartilage tissue was idealized as axis-symmetry, whereas the actual joint is not perfectly axis-symmetrical due to the presence of the lateral and medial meniscus and ligament. This limitation can be addressed in future studies by using data from CT and MRI-based 3D scans to construct a more realistic model geometry.

Thirdly, the loading condition applied in this study was modeled as a triangular distribution for simplicity. In vivo joint loading is more complex and often follows a parabolic distribution, involving a combination of compressive and shear forces. Moreover, this study did not simulate the transient load variations during the sit-to-stand transitions, which are known to contribute significantly to joint mechanics. Future studies should adopt more physiologically representative loading profiles, including dynamic transitions and mixed-mode forces, to improve the fidelity of the predicted transport behavior. By addressing these limitations, future research can provide a more biologically and mechanically accurate representation of IGF-1 transport and its regulatory effects in articular cartilage.

## Conclusion

This study developed a mathematical model to investigate the transport of IGF-1 through cartilage tissue under different ergonomic activities (e.g., STS) compared to the control case (free diffusion), accounting for the interaction between cartilage and the contact gap formed by surface asperities. Knee contact forces derived from musculoskeletal simulations were used to drive the model, capturing realistic workplace loading conditions that reflect both external posture and muscle-induced contributions. The aim was to examine the effect of workplace exercise on solute transport in articular cartilage, with particular focus on STS routines that align with WorkSafe Victoria’s recommendation for employees to take breaks after every 30 min of continuous sitting to reduce health risks. Accordingly, the modeled STS interventions reflect a range of feasible ergonomic workplace activities to evaluate their impact on cartilage solute transport. The key findings are summarized as follows:

- The effect of the contact gap and the associated fluid exchange between cartilage tissue and the gap plays a significant role in solute transport within cartilage. Neglecting the contact gap could lead to a substantial underestimation of the free, bound, and total solute concentrations.
- STS activities could significantly enhance solute transport compared to static sitting. For instance, $\:{t}_{si}={t}_{st}=10min$ results in approximately 15.8%, 9.6%, and 9.7% increases in free, bound, and total IGF-1 uptake ratios, respectively, after 5 h compared to static sitting.
- Reducing the loading interval could produce greater improvements in IGF-1 uptake. At $\:{t}_{si}={t}_{st}=10min$, IGF-1 uptake is 5–6% higher IGF-1 uptake across all categories than at $\:{t}_{si}={t}_{st}=30min$, demonstrating a clear dose-response relationship between activity loading duration and cartilage nutrient delivery.
- The beneficial effects of STS significantly emerge after the first hour of activity, when solute uptake in static sitting begins to plateau or decline. In contrast, STS conditions continue to promote increased uptake beyond the first hour, although the rate of improvement decreases over time.
